# Left ventricular diastolic dysfunction in pulmonary hypertension predicts functional capacity and clinical worsening: a tissue phase mapping study

**DOI:** 10.1186/s12968-015-0220-3

**Published:** 2015-12-29

**Authors:** Daniel S. Knight, Jennifer A. Steeden, Shahin Moledina, Alexander Jones, J. Gerry Coghlan, Vivek Muthurangu

**Affiliations:** Centre for Cardiovascular Imaging, UCL Institute of Cardiovascular Science, London, UK; UCL Medical School, Royal Free Campus, Rowland Hill Street, London, UK; Centre for Cardiovascular Imaging, UCL Institute of Cardiovascular Science, Institute of Child Health, 30 Guilford Street, London, WC1N 1EH UK

**Keywords:** Pulmonary hypertension, Diastolic dysfunction, Cardiovascular magnetic resonance

## Abstract

**Background:**

The function of the right and left ventricles is intimately related through a shared septum and pericardium. Therefore, right ventricular (RV) disease in pulmonary hypertension (PH) can result in abnormal left ventricular (LV) myocardial mechanics. To assess this, we implemented novel cardiovascular magnetic resonance (CMR) tissue phase mapping (TPM) to assess radial, longitudinal and tangential LV myocardial velocities in patients with PH.

**Methods:**

Respiratory self-gated TPM was performed using a rotating golden-angle spiral acquisition with retrospective cardiac gating. TPM of a mid ventricular slice was acquired in 40 PH patients and 20 age- and sex-matched healthy controls. Endocardial and epicardial LV borders were manually defined, and myocardial velocities calculated using in-house software. Patients without proximal CTEPH (chronic thromboembolic PH) and not receiving intravenous prostacyclin therapy (*n* = 34) were followed up until the primary outcome of disease progression (death, transplantation, or progression to intravenous therapy) or the end of the study. Physicians who determined disease progression were blinded to CMR data. Conventional ventricular volumetric indices and novel TPM metrics were analyzed for prediction of 6-min walk distance (6MWD) and disease progression.

**Results:**

Peak longitudinal (*p* < 0.0001) and radial (*p* = 0.001) early diastolic (E) wave velocities were significantly lower in PH patients compared with healthy volunteers. Reversal of tangential E waves was observed in all patients and was highly discriminative for the presence of PH (*p* < 0.0001).

The global radial E wave (β = 0.41, *p* = 0.017) and lateral wall radial systolic (S) wave velocities (β = 0.33, *p* = 0.028) were the only independent predictors of 6MWD in a model including RV ejection fraction (RVEF) and LV stroke volume.

Over a median follow-up period of 20 months (IQR 7.9 months), 8 patients commenced intravenous therapy and 1 died. Global longitudinal E wave was the only independent predictor of clinical worsening (6.3× increased risk, *p* = 0.009) in a model including RVEF and septal curvature.

**Conclusions:**

TPM metrics of LV diastolic function are significantly abnormal in PH. More importantly, abnormal LV E wave velocities are the only independent predictors of functional capacity and clinical worsening in a model that includes conventional metrics of biventricular function.

**Electronic supplementary material:**

The online version of this article (doi:10.1186/s12968-015-0220-3) contains supplementary material, which is available to authorized users.

## Background

Pulmonary hypertension (PH) is characterized by increased pulmonary artery pressure and right ventricular (RV) failure. Cardiovascular magnetic resonance (CMR) is the reference standard method of assessing RV function and is now routinely used in PH. Several studies have shown that CMR-derived RV volumes and ejection fraction (EF) are prognostic in this condition [1, 2].

It has also been shown that left ventricular ejection fraction (LVEF) is reduced in late stage PH [3]. However, the majority of PH patients have normal LVEF, and LV function is not prognostic [2]. Nevertheless, it is likely that these patients do have abnormal LV mechanics due to ventricular interdependence [4–8]. This could result in additional functional deficits, as is the case in patients with RV failure due to congenital heart disease [9]. Consequently, assessment of LV myocardial mechanics may be clinically useful in this patient population.

The most comprehensive way of evaluating LV mechanics is to assess the regional and geometric components of LV motion. There are several CMR methods that can be used to assess these metrics. In this study, we used tissue phase mapping (TPM) to assess radial, longitudinal and tangential myocardial velocities in patients with PH [10–13]. As this type of evaluation has not been performed in this population before, it is of uncertain clinical value. Therefore, our general aim was to explore the utility of TPM measures of LV myocardial mechanics in patients with PH.

The specific aims of this feasibility study were: i) to assess global and regional LV myocardial mechanics in healthy volunteers and in patients with PH with preserved LVEF, ii) to determine the relationship between myocardial velocities and exercise capacity, and iii) to test the ability of myocardial velocities to predict clinical worsening.

## Methods

### Study population

The study population consisted of 40 consecutive patients with PH and 20 healthy volunteers. Inclusion criteria for patients were: i) PH diagnosed by right heart catheterization (mean pulmonary artery pressure (mPAP) >25 mmHg and pulmonary capillary wedge pressure (PCWP) <15 mmHg) [14]; or ii) presentation for routine out-patient clinical evaluation with known PH, and/or right heart catheterization for diagnosis or follow-up of PH. Exclusion criteria for patients were: i) left-sided cardiac disease unrelated to PH (including ischemic heart disease, LV dysfunction or hypertrophy, and left-sided valve disease); ii) clinically significant restrictive or obstructive lung disease identified by pulmonary function tests; iii) arrhythmia; or iv) contraindications to CMR. Exclusion criteria for healthy volunteers were: i) past medical history of cardiovascular disease (including hypertension); ii) history of cardiac medications; iii) arrhythmia; or iv) contraindications to CMR.

All patients underwent the CMR study between 3rd October 2012 and 24th November 2013. All participants were imaged using a 1.5T MR scanner (Magnetom Avanto, Siemens Healthcare, Erlangen, Germany). All patients underwent a 6-min walk distance test (6-MWD). Twenty-nine patients (73 %) underwent clinically indicated right heart catheterization within 40 days of CMR (median 8 days, IQR 12 days).

The local institutional research ethics committee (North West London REC 2) approved the study and informed written consent was obtained from all participants.

### Conventional CMR protocol and image processing

Biventricular volumetric data were obtained as described previously, using a radial *k*-*t* SENSE real-time sequence [15], with contiguous transaxial and short-axis ventricular stacks acquired for RV and LV analyses respectively. Through-plane flow data were acquired in the ascending aorta, right and left branch pulmonary arteries, and for mitral valve inflow, using a velocity-encoded, prospectively-triggered spiral PCMR sequence [16].

All images were processed using in-house plug-ins for the open source OsiriX DICOM software platform (OsiriX Foundation, Geneva, Switzerland) [17]. Endocardial borders were traced manually at end-diastole and end-systole of both ventricles to assess biventricular function. This allowed evaluation of end diastolic volume (EDV) and end systolic volume (ESV), and calculation of stroke volume (SV) and ejection fraction (EF) [15]. Aortic and pulmonary artery flow were measured from the PCMR data, which were segmented using a semi-automatic vessel edge detection algorithm with manual operator correction [18]. Transmitral E and A wave peaks were measured from the mitral valve inflow PCMR data, allowing calculation of E/A ratio. Septal curvature was assessed using the midventricular short axis cine images, as described previously [19].

### Tissue phase mapping protocol and image processing

Myocardial velocities were acquired using a respiratory self-navigated, cardiac gated, velocity encoded golden-angle spiral sequence [13]. To summarize, a *two*-*sided* flow-encoding scheme (with positive and negative bipolar pulses applied for each velocity-encoding direction) was used to enable high temporal-resolution imaging (rather than conventional one-sided flow-encoding where four flow-encoded readouts are required). Data were continuously acquired, with each consecutive flow-encoding couplet rotated by the golden-angle. Consecutive spiral pairs (10 in each window) are combined to produce low temporal resolution (315 ms) real-time images. These real-time data are used to create an image based respiratory navigator, used to select 30 % of the expiratory spiral interleaves for the final retrospectively cardiac-gated reconstruction. Sequence parameters: TE/TR 3.85/14.9 ms, FOV 450 mm, Matrix: 384 × 384, uniformly distributed spiral interleaves required to fill k-space: 30 (for each of the three phase-encoded directions), slice thickness: 7 mm, VENC: 30 cm/s, Flip angle: 25°, pixel bandwidth: 930 Hz/pixel. This achieved a temporal resolution of 27.14 ms, with a spatial resolution 1.17 × 1.17 mm, giving approximately 40 cardiac phases. The nominal scan time, assuming a heart rate of 60 bpm and 100 % respiratory efficiency, would be 1 min 30 s, resulting in a scan time of approximately 4 to 5 min per subject depending upon heart rate. TPM data were acquired in mid-ventricular short-axis, which was chosen by reference to a 4-chamber cine at end-systole.

This sequence does not include any black blood pulses (as conventionally used in TPM) as this would have disrupted the continuous acquisition of data necessary for calculation of the respiratory navigator and retrospective cardiac gating. No off-resonance correction was performed as this would have increased the scan time or reduced the temporal resolution of the scan. Some minor image blurring was observed around fat tissue, but this did not severely affect the velocity measurements as the fat was generally spatially separated from the myocardium. Background phase offsets were minimized in the TPM data by optimizing the flow gradients and correcting for Maxwell terms. This resulted in no observable background phase offsets in the data.

All images were processed using an in-house plug-in for OsiriX [17]. For each dataset, endocardial and epicardial ventricular borders were manually segmented on the magnitude images to create a ventricular region of interest (ROI). The ventricular ROI was further split into four segments: septal, anterior, lateral and inferior. Bulk motion correction was performed [20], before transformation of the in-plane velocities to an internal polar coordinate system positioned at the center-of-mass of the LV. This allowed motion to be described in terms of radial (V_rad_), tangential (V_tang_) and longitudinal (V_long_) velocities [21]. For each direction, global velocities were calculated by averaging the velocity in a given direction (radial, longitudinal and tangential) within the ventricular ROI in each frame. Regional velocities were calculated by averaging the velocities in each segment. Peak systolic (S wave) and early diastolic (E wave) values were quantified from the longitudinal, radial and tangential velocity-time curves. The tangential S (S1 and S2) and E (E1 and E2) wave peaks were biphasic. Vector field plots and color-coded position-time maps were generated for each myocardial velocity component to allow easy visualization of the results.

### Statistics

STATA 13 was used for statistical analyses. Data were examined for normality using the Shapiro-Wilk test. Descriptive statistics are expressed as mean ± standard deviation (SD) when normally distributed and median (inter-quartile range, IQR) when non-normally distributed. Proportions are expressed as percentages.

Independent samples t-tests with Welch’s correction for unequal variances were used to compare parametric data in PH patients and controls (*n* = 11). The Mann–Whitney-*U* test was used for non-parametric data (*n* = 9). Fisher’s exact test was used to compare proportions data (*n* = 3). For subgroup analysis, PH patients were divided into 3 groups: PH associated with connective tissue disease (CTD), PH not associated with CTD, and chronic thromboembolic PH (CTEPH). The Kruskal-Wallis test was used to test for equality of abnormal global myocardial velocities (*n* = 5) between the different sub-groups of PH. The group of tests comparing normal controls to patients was considered a single family of statistical inferences and the familywise error rate was controlled using Bonferroni correction. Specifically, we adjusted for 28 statistical comparator tests resulting in a corrected critical *p*-value of <0.0018.

Random-effects generalized least squares models were used to compare myocardial velocities in the four myocardial segments. Interaction terms for the myocardial segment and presence of disease were included in the models. This analysis was only performed if the global velocities were abnormal (E_rad_, E_long_, S2_tang_, E1_tang_, and E2_tang_). In addition, this analysis was used to assess the timing of the E_rad_ peak, which on visual inspection appeared to vary between segments. Bonferroni correction was required to control the familywise error rate in this group of 6 generalized least squares models, and the adjusted critical *p*-value was 0.0083.

To assess the relationships between abnormal myocardial velocities and hemodynamic parameters a 2-stage procedure was employed. Firstly, simple univariate analysis was performed using Pearson’s correlation coefficient. This allowed selection of the conventional CMR biventricular parameters and afterload metrics with the strongest correlation to the abnormal myocardial velocities. To identify independent predictors of myocardial velocity these variables were entered into random-effects generalized least squares models. Variables in this model with a *p*-value of <0.05 were considered statistically significant.

A similar 2-stage analysis was performed to assess the relationship between 6-MWD and CMR data. From the univariate analysis, the strongest correlating E and S wave peaks and conventional CMR metrics were identified. These were then entered into a multiple linear regression model to determine covariates that were independently associated with 6-MWD. Variables in this model with a *p*-value of <0.05 were considered statistically significant.

All patients were followed up until death, transplantation, progression to intravenous epoprostenol, or the end of the study (February 25th, 2015). The decision to list a patient for transplantation or commence intravenous vasodilator therapy was based upon clinical assessment of deterioration in functional class and/or cardiac catheterization derived hemodynamic data. CMR data were not used in these management decisions and the physicians involved in the patients’ care were blinded to the CMR results. Univariate Cox proportional hazards analysis was used to assess the predictive ability of all CMR variables in the 34 patients without proximal CTEPH and not treated with intravenous epoprostenol. The primary outcome was freedom from death, transplantation or progression to intravenous therapy. The E and S waves and conventional CMR metrics with the greatest hazard ratios were entered into a multivariable Cox proportional hazards analysis to determine which covariates were independent predictors of clinical worsening. A *p*-value of <0.05 was taken as statistically significant.

## Results

### Study population characteristics

There was no difference in the age distribution of PH patients and normal controls (50 years (IQR: 45–59 years) versus 47 years (IQR: 42–54 years) respectively, *p* = 0.30). Thirty-out-of-forty patients were female compared to 16 out of 20 controls (*p* = 0.76). Patient characteristics and underlying diagnoses are detailed in Table 1. The largest patient sub-group had PH associated with CTD (20/40), the next largest sub-group (12/40) had PH not associated with CTD (10/12 had idiopathic PAH), followed by patients with CTEPH (8/40). All patients were normotensive at the time of the study with a median systolic BP of 110 mmHg (IQR: 105–120 mmHg) and a mean diastolic BP of 72 ± 11 mmHg. Thirty-two patients were receiving PH therapy at the time of CMR study (Table 1).

**Table 1:** Patient characteristics

Etiology [33]	Number (%)
1. PAH	11 (28)
- 1.1 Idiopathic	10
- 1.2 Heritable	1
1.4 Associated with (APAH):	21 (53)
- 1.4.1 CTD	20
- Limited cutaneous systemic sclerosis	13
- Diffuse cutaneous systemic sclerosis	2
- Systemic lupus erythematosus	3
- Sjögren’s syndrome	1
- Mixed CTD	1
- 1.4.3 Portal Hypertension	1
4 CTEPH	8 (20)
- Proximal (operable)	5
- Distal (inoperable)	3
Right Heart Catheterization (RHC) data (*n* = 29)	
Time interval between CMR and RHC (days)	8 (IQR 12)
Mean pulmonary artery pressure (mPAP, mmHg)	46 ± 13
Pulmonary vascular resistance (PVR, dyn · s/cm^5^)	523 (IQR 402–717)
World Health Organization class (%)	
I	1 (2.5)
II	14 (35)
III	22 (55)
IV	3 (7.5)
6-MWD (metres)	372 (IQR 286–450)
Vasodilator therapy	
Phosphodiesterase type 5 inhibitor	28 (70)
Endothelin receptor antagonist	22 (55)
Intravenous prostacyclin	1 (3)
Treatment naive	8 (20)
Oral monotherapy	14 (35)
Dual combination oral therapy	16 (40)
Triple combination therapy	1 (3)
Additional PH therapies	
Oral prostacyclin	1 (3)
Inhaled prostacyclin	1 (3)
Tyrosine-kinase inhibitor	2 (5)

Conventional CMR metrics from normal subjects and PH patients are shown in Table 2. In PH, the RV was dilated with reduced RVEF and the LV was compressed with reduced SV. In addition, septal curvature was lower (or reversed in 65 % of patients). Nevertheless, LVEF and E/A ratio were not significantly different between patients and controls.

**Table 2:** CMR characteristics of control subjects versus patients with PH

	Normal	PH patient	*p*-value
RVEDV (mL)	130 (104–156)	158 (128–204)	0.0056
RVESV (mL)	38 (31–63)	103 (70–133)	<0.00001*
RVSV (mL)	87 (74–99)	62 (48–75)	0.0006*
RVEF	65 ± 8	40 ± 12	<0.00001*
LVEDV (mL)	134 (108–139)	88 (74–109)	0.0001*
LVESV (mL)	42 ± 16	32 ± 13	0.012
LVSV (mL)	85 ± 19	62 ± 18	<0.00001*
LVEF	67 ± 8	66 ± 9	0.61
LV cardiac output (L/min)	5.6 (4.8–6.4)	4.6 (3.8–5.5)	0.0094
Septal curvature	1.09 ± 0.10	−0.20 ± 0.58	<0.00001*
E/A ratio	1.56 (1.23–2.88)	1.33 (1.11–2.36)	0.31

Values are mean ± SD, or median (interquartile range)

*indicates statistical significance (*p* < 0.0018 following Bonferroni correction)

Median 6-MWD in PH patients was 372 m (IQR: 286–450 m). Twenty-nine PH patients underwent clinically indicated right heart catheterization within 40 days of CMR (median 8 days, IQR 12 days). Median pulmonary vascular resistance (PVR) was 523 dyn · s/cm^5^ (IQR 402–717 dyn · s/cm^5^) and average mPAP was 46 ± 13 mmHg. All patients had a PCWP < 15 mmHg.

### Myocardial velocities in normal subjects and PH patients

**Fig. 1:**
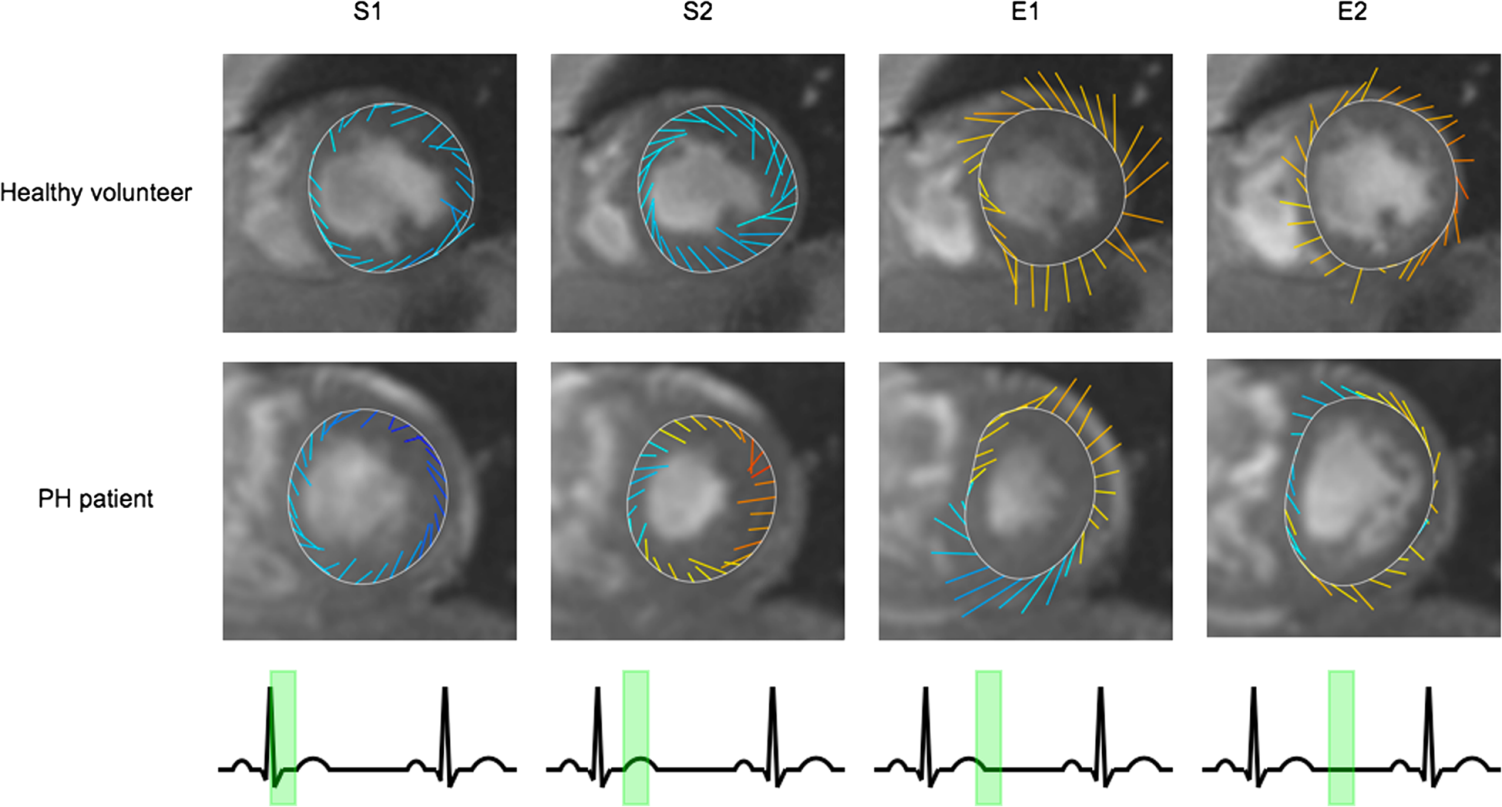
LV velocity vector plots from a healthy volunteer and a PH patient at four time points in the cardiac cycle (indicated by the *ECG trace*). The line colors represent longitudinal velocities: *blue* or *yellow/red* represent myocardial motion towards or away from the apex respectively. The line orientation and length represents the vector sum of the radial and tangential velocities. Note the biphasic tangential systolic (S1 and S2) and early diastolic (E1 and E2) motions. In health, this follows an anti-clockwise then clockwise motion in systole and diastole. In PH, however, there is reversal of early diastolic tangential untwisting directions: clockwise untwisting occurs prior to anti-clockwise untwisting

Myocardial velocities were acquired successfully in all subjects. Figure 1 shows representative LV velocity vectors in a normal subject and a PH patient.

**Fig. 2:**
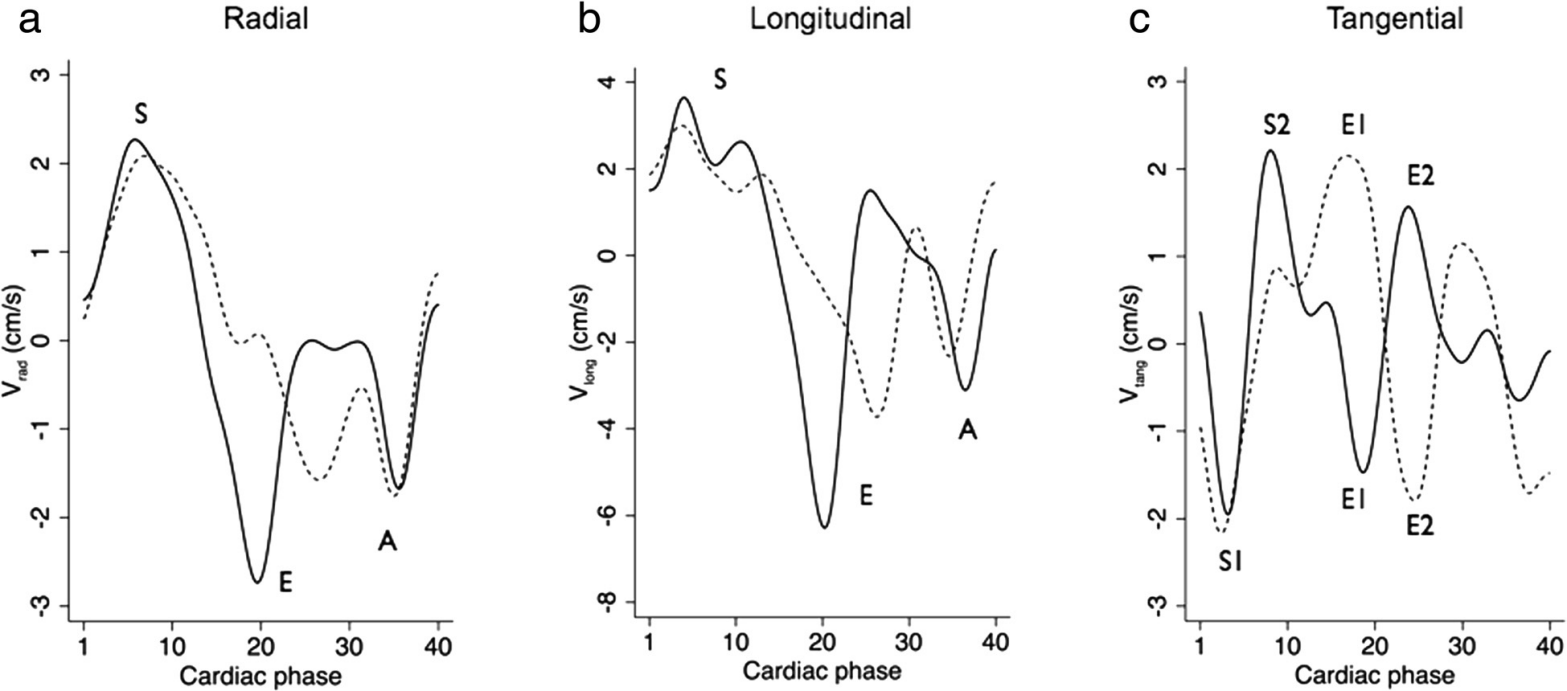
Line graphs of global **a** radial, **b** longitudinal and **c** tangential LV velocities in a healthy volunteer (*solid line*) and a PH patient (*dotted line*). The RR intervals for both subjects have been scaled to the same value for illustrative purposes. S, E and A waves are evident radially and longitudinally. Biphasic systolic (S1 and S2) and early diastolic (E1 and E2) waves are observed tangentially. Cardiac cycles are normalized for heart rate for illustrative purposes. Global E_rad_ and E_long_ velocities are markedly reduced in PH. The tangential S2 wave is also reduced in PH. The reverse direction of untwisting of biphasic early diastolic tangential waves was evident in all PH patients

Global myocardial peak velocities in normal subjects and PH patients are summarized in Table 3. Patients with PH had reduced LV peak E_rad_, E_long_ and S2_tang_ velocities, with reversal and significant change in both E_tang_ peaks. This can be appreciated in Fig. 2, which shows representative global radial, longitudinal, and tangential velocity curves from a normal subject and a PH patient. Reversal of both E_tang_ waves was highly discriminatory for the presence of PH. All patients had a reversed E1_tang_ compared to 4/20 normal subjects (*p* < 0.0001), while 32/40 patients had a reversed E2_tang_ compared to 2/20 normal subjects (*p* < 0.0001). The magnitude of all abnormal global myocardial velocity peaks did not differ between etiological groups (*p* > 0.59).

**Table 3:** Peak global myocardial velocities in control subjects versus patients with PH

Global velocity	Normal (cm/s)	PH patient (cm/s)	*p*-value
LV S_rad_	2.4 ± 0.4	2.3 ± 0.5	0.14
LV E_rad_	3.1 ± 0.5	2.3 ± 1.0	0.001*
LV S_long_	3.3 ± 1.0	3.3 ± 1.1	0.98
LV E_long_	4.2 (3.4, 5.5)	2.6 (1.7, 3.3)	<0.00001*
LV S1_tang_	−1.8 ± 0.7	−1.4 ± 0.8	0.064
LV S2_tang_	1.3 ± 0.6	0.50 ± 0.69	0.0001*
LV E1_tang_	−0.4 ± 0.6	1.4 ± 0.5	<0.00001*
LV E2_tang_	0.9 (0.7,1.3)	−0.8 (−1.1, −0.3)	<0.00001*

Values are mean ± SD, or median (interquartile range)

*indicates statistical significance (*p* < 0.0018 following Bonferroni correction)

### Regional variations in normal subjects and PH patients

**Fig. 3:**
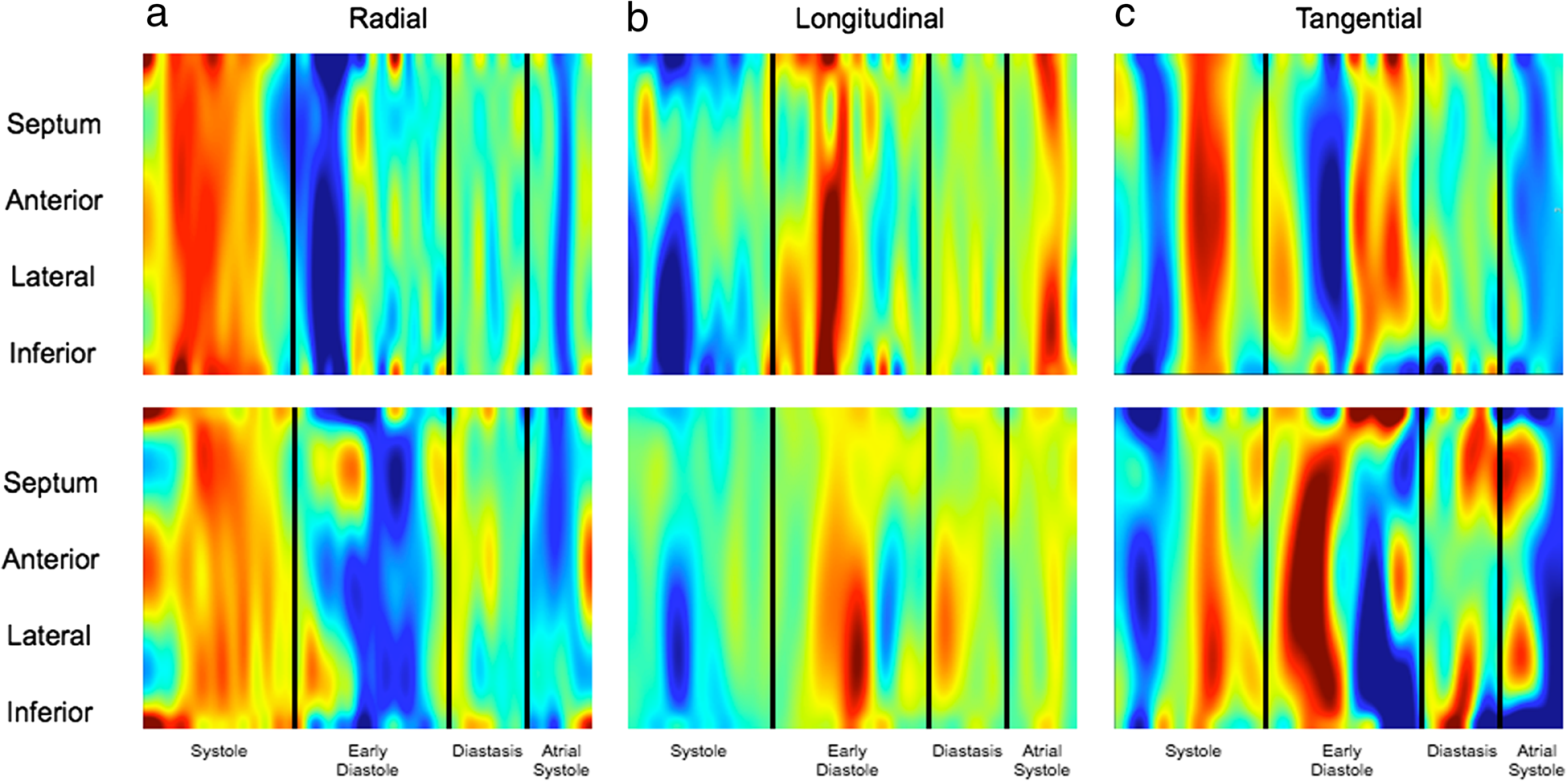
Velocity color maps for **a** radial, **b** longitudinal, and **c** tangential segmental LV motion from a healthy volunteer (*top*) and a PH patient (*bottom*). The maps represent motion of sequential LV segments (y-axis) throughout the cardiac cycle (x-axis). The wave color (*blue* or *red*) indicates direction of motion, with color intensity representing relative magnitude of the segmental velocity. There are segmental E wave abnormalities in all components of motion: **a** Radially: In health, E wave timing is uniform throughout LV segments. In early diastole in PH, the anterior segment tends to move outwards (E_rad_ wave) first whilst the septum continues to move inwards. **b** Longitudinally: In health, peak anterolateral segment E_long_ waves are of significantly greater magnitude, compared with only a trend for greater lateral segment E_long_ waves in PH. **c** Tangentially: Reversal of E1 and E2 waves was observed in all patients

**Fig. 4:**
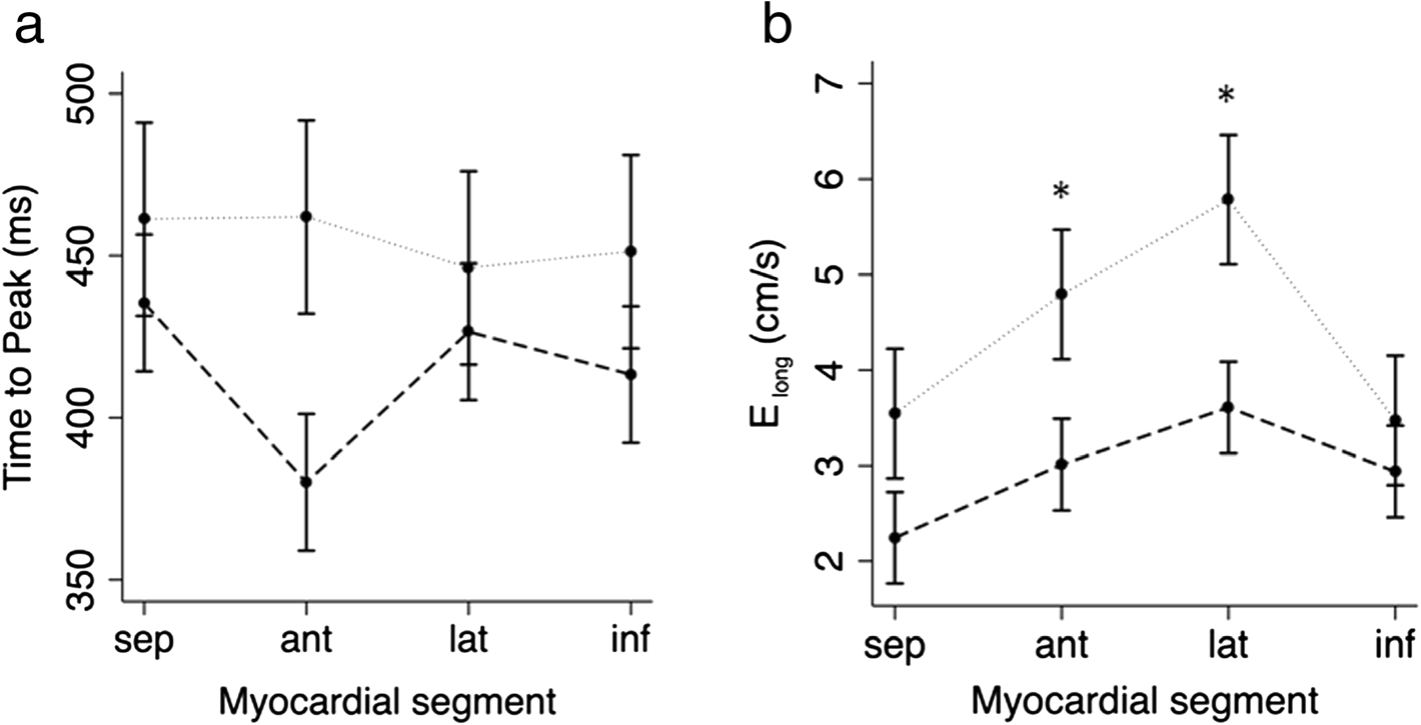
Graphs demonstrating segmental variation in health (*dotted line*) and PH (--- *line*) in **a** E_rad_ time to peak, **b** E_long_ magnitude. **a** There is a trend for an earlier E_rad_ time to peak for the anterior segment in PH. There is no regional heterogeneity in E_rad_ time to peak in health. **b** The anterior and lateral walls had higher E_long_ peak velocities than the inferior wall and septum in health. Regional variation in the magnitude of E_long_ velocities is less distinct in PH, with only the lateral segment having a tendency, albeit not statistically significant, for higher E_long_ velocities

Figure 3a shows radial LV myocardial velocities as a function of position and time in a normal subject and a patient with PH. In patients, there was a trend towards E_rad_ velocity peaking in the anterior segment first (approximately 80 ms, *p* = 0.026) as seen in Fig. 4. However, there was no significant regional difference in the magnitude of the E_rad_ peaks (*p* > 0.07).

Longitudinal velocity maps are shown in Fig. 3b. In normal subjects, E_long_ peak velocity was similar in the inferior wall and septum (*p* = 0.88) but higher in the anterior (*p* = 0.008) and lateral walls (*p* < 0.001). However, in patients, this regional variation was less distinct (Fig. 4) with only a trend towards higher E_long_ peak in the lateral segment (*p* = 0.012).

Figure 3c shows tangential velocity maps in a normal subject and patient. There were no regional variations in tangential velocity peaks in controls or patients.

### Hemodynamic correlates with myocardial velocities

Univariate hemodynamic correlates with abnormal global myocardial velocities are shown in Table 4. Both peak E_rad_ and E_long_ strongly correlated with mPAP, RVESV and E/A ratio. In addition, peak E_rad_ also correlated with LVSV. The first global E_tang_ peak only correlated with RVESV (r = 0.35, *p* = 0.023). The second E_tang_ and S_tang_ peaks did not correlate with any of the tested metrics (*p* > 0.1). In a generalized least squares model that included mPAP, RVESV, LVSV and E/A, only RVESV was independently predictive of either longitudinal or radial E wave velocities (β = −0.43, *p* = 0.001).

**Table 4:** Correlations between abnormal global LV myocardial velocities in PH and hemodynamics

	E_rad_		E_long_		S2_tang_		E1_tang_		E2_tang_	
	r	*p*-value	r	*p*-value	r	*p*-value	r	*p*-value	r	*p*-value
SC	0.40	0.01	0.33	0.041	−0.041	0.80	−0.24	0.13	0.13	0.43
mPAP	−0.60	0.0005	−0.41	0.027	0.17	0.38	0.17	0.38	−0.19	0.32
PVR	−0.47	0.011	−0.27	0.16	0.046	0.82	0.14	0.49	−0.16	0.43
RVEDV	−0.26	0.11	−0.41	0.0087	0.0024	0.99	0.24	0.14	0.071	0.66
RVESV	−0.45	0.004	−0.51	0.0007	0.030	0.86	0.35	0.023	0.062	0.71
RVSV	0.33	0.038	0.1006	0.54	−0.074	0.65	−0.17	0.29	0.040	0.81
LVEDV	0.34	0.035	0.13	0.43	0.083	0.61	−0.18	0.27	0.20	0.22
LVESV	0.061	0.71	−0.035	0.83	0.24	0.13	−0.093	0.57	0.29	0.067
LVSV	0.45	0.0032	0.22	0.18	−0.063	0.70	−0.20	0.23	0.077	0.64
E/A ratio	0.43	0.0077	0.38	0.019	−0.016	0.93	0.031	0.85	−0.0035	0.98

### Functional correlates with myocardial velocities

All correlates with 6-MWD are shown in Additional file 1: Table S1. The strongest E wave myocardial velocity correlate with 6-MWD was the global E_rad_ peak (r = 0.58, *p* = 0.0001). The strongest S wave myocardial velocity was the lateral S_rad_ peak (r = 0.48, *p* = 0.0018). The conventional RV metric with the strongest correlation to 6-MWD was RVEF (r = 0.54, *p* = 0.0003) and the strongest conventional LV metric was LVSV (r = 0.39, *p* = 0.013). In addition, there was a significant correlation between 6-MWD and E/A ratio (r = 0.39, *p* = 0.013). When these metrics were inputted into a multiple linear regression analysis, only global E_rad_ (β = 0.41, *p* = 0.017) and lateral S_rad_ (β = 0.33, *p* = 0.028) were independent predictors of 6-MWD.

### Relationship between myocardial velocity and clinical worsening

All five patients who had operable proximal CTEPH underwent subsequent thromboendarterectomy, and one PAH patient was already receiving intravenous vasodilator therapy at the time of CMR study. Clinical worsening was studied in the remaining 34 patients. Over a median follow-up period of 20 months (IQR 7.9 months), 8 patients were started on intravenous therapy and 1 died.

Metrics that predicted clinical worsening on univariate cox regression analysis are shown in Additional file 2: Table S2. The strongest predictive E wave myocardial velocity was global E_long_ peak (6.3× increase in hazard per SD reduction in the magnitude of the peak velocity). The strongest conventional RV metric was RVEF (2.4× increase in hazard per SD reduction in RVEF). In addition, septal curvature was also predictive of clinical worsening (3.6× increase in hazard per SD distortion of septum towards the LV). Conventional LV metrics and E/A ratio did not predict clinical worsening. When global E_long_ peak, RVEF and SC were inputted into a multivariable cox regression model, only global E_long_ was an independent predictor of clinical worsening (*p* = 0.009).

## Discussion

This is the first study to use TPM to assess LV myocardial velocities in patients with PH. The main findings were: i) Patients with PH had reduced global E_rad_ and E_long_ velocities, and reversal of both E_tang_ waves; ii) Peak global E_rad_ velocity was an independent predictor of 6-MWD; and iii) Peak global E_long_ velocity was an independent predictor of clinical worsening. These results demonstrate that LV myocardial mechanics are negatively affected by RV pressure overload and may contribute to symptoms and clinical worsening.

### LV myocardial velocities in PH

In keeping with previous studies, our results indicate that PH is primarily associated with early diastolic LV dysfunction [5, 8, 22]. Specifically, peak E wave velocities were lower in patients compared to age and sex matched controls. Importantly, LVSV was not an independent predictor of E wave velocities. Thus, it is unlikely that reduced pulmonary venous return is the main reason for this finding. Interestingly, the only independent predictor of reduced E wave velocity was increased RVESV. This suggests a link between RV dilation and LV diastolic dysfunction, which we believe is mediated through external constraint of the LV. It is easily understood that LV filling can be reduced by abnormal septal dynamics [9]. However, it is also possible that the pericardium also plays an important part. As the RV dilates, the whole of the pericardium becomes stretched and less compliant [23, 24]. This could constrain the inferior and lateral LV walls and additionally reduce LV filling. This idea is backed up by animal studies of acute RV dilation, where removal of the pericardium normalizes LV filling [25, 26]. The fact that the anterior segment is not constrained by the pericardium or septum may explain the trend towards its earlier E_rad_ peak, further corroborating our hypothesis. The exact mechanism underlying the reversal of the E_tang_ peaks is not clear from our results. A possible explanation might be the significant geometric alterations seen in patients with PH, but this requires further study.

It should be noted that these diastolic abnormalities could be the result of LV remodeling and intrinsic myocardial stiffening. This is particularly pertinent in our patient population due to the high prevalence of CTD, which is known to cause diastolic dysfunction [27]. However, E wave velocities were similar across etiological subgroups in our study, suggesting that the results were not due to CTD-specific LV remodeling. It is possible that PH itself could cause changes in LV structure such as myocardial fibrosis or fiber reorientation [28]. Techniques such as T1 Mapping and myocardial diffusion tensor imaging may be better placed to determine if these factors are also important [29].

### Functional correlates with myocardial velocities

We have shown that lower radial and longitudinal E wave velocities are associated with reduced 6-MWD. This is in keeping with E wave velocities being a marker for diastolic dysfunction, which is known to limit augmentation of stroke volume during exercise. In keeping with the pivotal role of stroke volume, it is unsurprising that resting LVSV also correlated with 6-MWD. However, global E_rad_ was an independent predictor of 6-MWD in a model adjusted for resting LVSV. This suggests that resting E_rad_ may be a better predictor of exercise stroke volume augmentation than resting LVSV. In addition, the lateral S_rad_ peak was also predictive of 6-MWD. This is interesting because the population S_rad_ peaks were similar in patients and controls. Nevertheless, patients did have greater variance in S_rad_, which may explain the exercise findings. The increased peak systolic velocity seen in some patients is probably an attempt to maintain cardiac output in the face of worsening disease. Conversely, the reduced peak velocity found in other patients is possibly due to intrinsic LV systolic dysfunction or abnormal septal interactions.

We also found that E wave velocities, in particular global E_long_, predicted clinical worsening. This is probably because patients with impaired diastolic function have less cardiac reserve and are therefore more symptomatic. This increases the likelihood of up-titration of therapy or death. The reasons why longitudinal rather than radial E wave is a better predictor of progression are not obvious from our data. One possibility is that longitudinal velocities might integrate more measures of cardiac dysfunction than simply reduced LV filling. Importantly, E/A ratio was similar in patients and controls and did not independently predict 6-MWD or clinical worsening. This demonstrates the benefits of TPM over conventional measures of diastolic dysfunction.

In keeping with previous studies, RVEF did correlate with 6-MWD and predicted clinical worsening [1]. However, RVEF was not an independent predictor of exercise capacity in a model including global E_rad_, nor was it an independent predictor of clinical worsening in a model adjusted for global E_long_. These results suggest that reduced LV diastolic function may be more important than RV function itself. This is consistent with studies in patients with other forms of RV pressure overload. For instance, in congenital heart disease it has been shown that improved exercise capacity after relief of RV outflow obstruction is primarily due to better LV filling [9].

### Assessing myocardial motion using imaging

We used TPM to assess myocardial mechanics. However, other CMR methods of assessing myocardial motion do exist. These include tagging, strain encoding (SENC) and displacement encoding with stimulated echoes (DENSE) [30]. Although these techniques have the benefit of measuring strain rather than velocity, they do have limitations. For instance, tagging does not analyze through plane motion, while SENC imaging is unable to measure radial strain. In contrast, high spatiotemporal resolution TPM allows the measurement of simultaneous, multi-directional velocity encoded data acquired throughout the entire cardiac cycle [12, 13]. This allows accurate analysis of global and regional myocardial velocities and timing parameters. Recently, it has been shown that it is possible to acquire TPM data in a breath hold using a spiral SENSE acquisition [31]. This opens up the possibility of rapid acquisition of myocardial velocities, which would make this technique more clinically feasible.

## Limitations

Our feasibility study represents a single center experience of applying TPM to a small patient cohort. Furthermore, the low mortality in this population required a more broadly defined composite outcome measure that included transplant and intravenous therapy. Unfortunately, such ‘soft’ outcome measures are more susceptible to bias (although CMR was not used to make clinical management decisions in this study). In addition, the population was heterogeneous with a high number of patients with CTD, possibly limiting the applicability of the results to the majority of PH patients. Therefore, this can only be considered a feasibility study demonstrating that TPM data may be of clinical interest in this group of patients. Nevertheless, the positive findings do warrant further work in this area.

Other limitations include the fact that catheter hemodynamic data was not available in all patients and that formal tissue characterization was not performed. In future studies, it will be vital that these deficiencies are addressed.

A final important limitation is that RV TPM metrics were not assessed in this study. It has been shown that it is possible to assess RV TPM metrics using our technique [13]. However, a limitation of short axis TPM is that it is not possible to correct for longitudinal bulk motion. In the LV this is not a significant problem as longitudinal bulk motion is limited. However, in severe PH the RV displays a rocking motion that results in errors in longitudinal velocity assessment [32]. Thus, assessment of RV myocardial velocities would be better achieved in the 4-chamber view and this would be important in future work.

## Conclusions

Novel TPM by CMR is feasible in PH, permitting accurate quantification of global and regional myocardial velocities. TPM metrics of LV diastolic dysfunction in PH reliably discriminate between health and disease, and are also strongly predictive of functional capacity. TPM may also be incrementally beneficial in identifying clinical worsening in PH compared with conventional CMR metrics of RV function. These feasibility data support the application of the technique to a larger group of patients over a longer follow-up period. This would allow full determination of the prognostic capacity of LV TPM metrics in PH. Future work should also be directed at assessing the response of these novel biomarkers to vasodilator therapy.

## Additional files

Additional file 1: Table S1. Correlates of conventional and novel CMR metrics with 6-MWD in PH. (XLSX 42 kb) Additional file 2: Table S2. Univariate Cox regression analysis of conventional and novel CMR metrics to predict disease progression in PH. (XLSX 44 kb)

## Abbreviations

6-MWD: 6-min walk distance test
CMR: Cardiovascular magnetic resonance
CTD: Connective tissue disease
CTEPH: Chronic thromboembolic pulmonary hypertension
EDV: End diastolic volume
EF: Ejection fraction
ESV: End systolic volume
LV: Left ventricle
PH: Pulmonary hypertension
RV: Right ventricle
SV: Stroke volume
TPM: Tissue phase mapping
SD: Standard deviation
